# False positive result of human chorionic gonadotropin caused by human anti-mouse antibodies

**DOI:** 10.11613/BM.2023.010802

**Published:** 2023-02-15

**Authors:** Jaroslav Racek, Ivana Potočová, Daniel Rajdl, Ladislav Trefil, Marie Šolcová

**Affiliations:** 1 Department of Clinical Biochemistry and Hematology, University Hospital and Faculty of Medicine, Charles University, Pilsen, Czech Republic; 2 Department of Gynecology and Obstetrics, Klatovska Hospital, Klatovy, Czech Republic

**Keywords:** anti-mouse antibodies, false positivity, hCG, interferences, pregnancy

## Abstract

Immunochemical reactions are fast, can be automated, and generally do not require pretreatment of biological material. Based on these advantages, they are widely used. On the other hand, they are susceptible to analytical interference that can lead to inaccurate results. These factors include the presence of anti-mouse antibodies, causing false positive (or sometimes false negative) results. Although the anti-mouse antibodies over many decades have been repeatedly identified to be the causative source but due to the rarity of such encounters they remain insufficiently considered. Here we show a case, a 45 year-old female who was mis-diagnosed with pregnancy due to falsely elevated human chorionic gonadotropin (hCG) due to anti-mouse antibodies. This led to the patient undergoing two ultrasound examinations and laparoscopy before the hCG was repeated on alternative assays which showed negative results, preventing the patient from methotrexate treatment. Here we describe the details of the case, outline the assay principal, supporting the finding from literature and outlining a process on how to identify such interferences in timely manner.

## Introduction

Immunochemical reactions are used in clinical practice to determine antigens (plasma proteins, cardiac markers, concentrations of therapeutic drugs, hormones, vitamins, bone markers, tumor markers, *etc.*) and antibodies, including autoantibodies. They are fast, can be automated, and generally do not require pretreatment of biological material. However, we have to reckon with numerous analytical difficulties. These include the hook effect, given by the dissolution of the antigen–antibody complex in the excess of antigen, or cross-reactivity, that is, several related antigens react with the same antibody. Improper evaluation, the failure to recognize false negative or false positive results, can lead to misdiagnosis, inappropriate intervention or treatment, or, conversely, to its omission. To detect them, we must be aware of this possibility, and it is also necessary to know the principles of immunochemical methods; this is the task of experts in clinical chemistry.

Unexpected results can be caused by, for example:

the presence of complexes of the determined antigens with immunoglobulins (*i.e.,* with autoantibodies), such as in the case of macroenzymes, macrotroponin, macroprolactin, or macro-human chorionic gonadotropin (hCG) (*1*-*7*);
the presence of cross-reactive substances with an antibody against the antigen to be determined, which is a component of a reagent, such as digoxin-like immunoreactive substances (*8*-*10*);
the interference of biotin (used as a drug or dietary supplement) in kits based on the fixation of the immune complex to microparticles through binding of streptavidin to biotin (*11*-*13*);
the presence of antibodies to murine antigens when the kit uses murine antibodies (*14*-*16*).

The latter interference has been described repeatedly for the human chorionic gonadotropin (hCG) assay (*14*-*16*). Due to its significance and possible unfavorable consequences, it has earned the designation “phantom hCG” (*17*, *18*). The diagnosis of pregnancy is determined on the basis of physical examination, laboratory evaluation (the finding of hCG in urine and blood serum), and ultrasonography; hCG can in most cases serve as the first ‘’early’’ marker of pregnancy. The described case of a 45 year-old female who was mis-diagnosed with pregnancy due to falsely elevated hCG due to anti-mouse antibodies aims to show this important issue that is still overlooked, and at the same time provides guidance on how to proceed so that interference could be detected in time.

## Case report

A 45-year-old woman, in her fourth gravidity, was monitored by a practical gynecologist for amenorrhea (it had been 43 days since her last menses). Determination of hCG in urine was not performed, its concentration in blood serum fluctuated around 1450 U/L (reagent kit Total β-hCG, based on a chemiluminescent microparticle immunoassay (CMIA) technology on Architect i1000SR analyser (Abbott, Abbott Park, USA); however, ultrasound did not find a gestational sac. The patient was sent to a regional hospital with the diagnosis of pregnancy of unknown localization, to exclude ectopic pregnancy.

Here, due to a slight decrease in hCG concentration, a revision of the uterine cavity was performed with a negative finding. As there was another slight decrease in the hCG concentration the next day, the uterine cavity revision was repeated and supplemented with a laparoscopic examination; again, the gestational sac could not be found. Finally, the patient was transferred to the university hospital where methotrexate administration was considered. However, blood was first drawn for hCG determination. Its concentration was surprisingly < 1 U/L (reagent kit ELECSYS hCG+βb, based on electrochemiluminiscence (ECLIA), Cobas e602 analyser (Roche Diagnostics GmBH, Mannheim, Germany) in two different samples.

Since this result was quite different from the previous ones, one of the above mentioned samples was sent to two other laboratories to obtain results using kits from other manufacturers. The results were 2265 U/L on an Abbott Architect i1000SR and 4.2 U/L using reagent kit Access Total Beta-hCG, based on chemiluminiscence (CLIA) on Unicel DxI 800 Access Immunoassay System (Beckman Coulter, Brea, USA). Therefore, the presence of heterophilic anti-mouse antibodies was suspected. After incubation of the sample in heterophilic blocking tubes (HBT, Scantibodies Laboratory, Inc., Santee, USA), the hCG result was < 1.2 U/L on an Abbott Architect i1000SR. According to the producer, the HBT contains a unique blocking reagent composed of specific binders which inactivate heterophilic antibodies. Therefore, pregnancy was ruled out, and the patient was discharged to home care.

**Figure 1:**
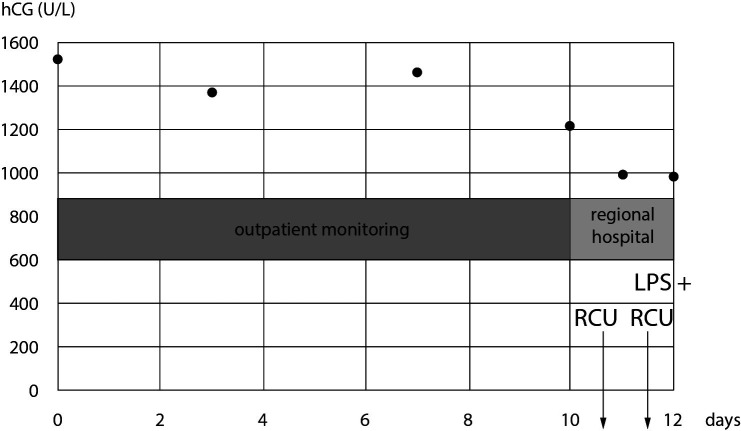
The course of human chorionic gonadotropin (hCG) concentrations and procedures performed during outpatient monitoring and the first hospitalization. LPS – laparoscopy. RCU - *revisio cavi uteri.*

Results of hCG measurement in the first sample taken in the university hospital using kits and analysers from different manufacturers are summarized in Table 1. The course of human chorionic gonadotropin (hCG) concentrations and procedures performed are summarized in Figure 1.

**Table 1:** Serum hCG concentration using kits and analysers from different manufacturers and the effect of pre-incubation in heterophilic blocking tubes (for all measurements, the first sample obtained during hospitalization at the university hospital was used)

**Reagent kit, instrument (company)**	**hCG (U/L)**
Total β-hCG, Architect i1000SR analyser (Abbott, Abbott Park, USA)	2265
Total β-hCG, Architect i1000SR analyser (Abbott, Abbott Park, USA), after pre-incubation in a heterophilic blocking tube	< 1.2
ELECSYS hCG+βb, Cobas e602 analyser (Roche Diagnostics GmBH, Mannheim, Germany)	< 1.0
Access Total Beta-hCG, Unicel DxI 800 Access Immunoassay System (Beckman Coulter, Brea, USA)	4.2
hCG - human chorionic gonadotropin.	

The authors have at their disposal the patient’s informed consent, which relates to the described case report.

## Discussion

The interference of heterophilic antibodies in the determination of hCG has been described repeatedly (*19*-*21*). The production of heterophilic antibodies in the body is caused by external (heterophilic) antigens. These include antibodies against animal antigens, most often against mouse antigens: human anti-mouse antibodies (HAMA). They occur in the persons having contact with mice – laboratory, veterinary, pets or as a result of animal products in the diet, or after the administration of certain animal proteins found in some imaging agents or therapeutic monoclonal antibodies produced in mice used for biological treatment of tumors and autoimmune diseases (*15*, *16*). They can significantly interfere with immunochemical assays (especially assays based on the sandwich principle), leading to false positive or false negative results.

**Figure 2:**
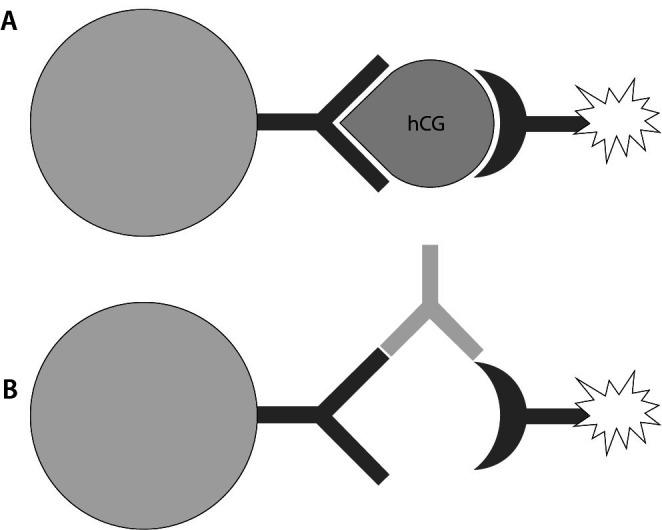
Principle of (A) determination of human chorionic gonadotropin (sandwich principle) and (B) human anti-mouse antibody (HAMA) interference causing a false positive result. See the text for further explanation.

The principle of immunochemical determination of hCG is shown in Figure 2a: hCG in the patient’s serum binds to mouse antibodies on the surface of magnetizable microparticles. Then, other mouse anti-hCG antibodies labeled with acridinium are added. Only a portion of these second antibodies binds to hCG, while the rest remains free. After washing, only bound labeled antibodies remain and the labeling is measured in a chemiluminescent reaction. Separation of unbound and bound antibodies is possible as a result of binding to magnetizable microparticles (*22*).

When HAMA are present in blood serum, they form a bridge between the two murine antibodies. After washing, chemiluminescence can be detected, resulting in a false positive result (Figure 2b). At other times, the presence of HAMA can lead to a false negative result: binding of HAMA to a second mouse antibody prevents its binding to the antigen of interest. Similarly, in a competitive immunoassay, the binding of HAMA to an antibody may prevent it from binding to the antigen of interest.

Most false positive hCG results are due to the presence of HAMA. Another possibility is interference from human luteinizing hormone (LH) and hCG originating from the pituitary gland; this should be considered especially in elderly patients and patients after adnexectomy (*17*). According to the manufacturer, the LH interference is < 10% (*22*). Finally, elevated hCG levels may be due to the presence of macro-hCG as a result of the binding of antibodies generated after stimulation with hCG prior to *in vitro* fertilization (*7*). Failure to recognize false positivity can lead to the indication and implementation of unnecessary diagnostic and treatment procedures (uterine cavity revision, laparoscopy, administration of cytostatic agents, or even hysterectomy or adnexectomy) (*17*, *19*). Prompt collaboration between clinicians and laboratory staff is essential to resolve such unexplainable results.

What should be suspicious when measuring hCG? We determine hCG with the aim of diagnosing:

pregnancy – the hCG concentration doubles in approximately 48 hours;
extrauterine pregnancy – the hCG concentration rises more slowly, but it still rises;
trophoblast tumor – the hCG concentration also increases;
dead fetus – the hCG concentration decreases.

Thus, in all cases, there is a dynamic change in the hCG concentration (*18*), which was missing at first in our patient, and later on there was a decrease in concentration, but it was relatively small.

How do we proceed if HAMA are suspected? Many authors have made recommendations on how to avoid an incorrect assessment of hCG levels (*23*-*26*). The American College of Obstetricians and Gynecologists, Committee on Gynecologic Practice, has even issued an opinion that addresses this problem (*27*).

The following paragraphs show the recommended procedure according to (*27*); we should proceed in the following order. The results of the individual steps are given in parentheses, if the cause of the false positivity of hCG in the blood serum is the presence of heterophilic antibodies.

1. Measure hCG in urine (the result is negative because the antibodies to mouse proteins do not enter the urine).
2. Try another manufacturer‘s kit that does not use mouse antibodies (the result is negative).
3. Try to gradually dilute the serum and determine hCG (linearity parallel to dilution is not found, that is, recovery is more than 100%).
4. Measure serum hCG after preincubation in HBT (the result is negative).

In our patient, the simplest and easily available procedure – the measurement of hCG in the urine – was unfortunately omitted at the very beginning because the possible interference was not taken into account.

Following this procedure, false positivity of hCG due to the presence of heterophilic antibodies can be detected. For this reason, every laboratory should have these blocking tubes available.

One last note on our patient: when she was 36 years old (9 years prior to the current case) she had been hospitalized at an internal medicine clinic for intermittent chest pain and elevated serum cTnI with a suspected acute coronary event. The examinations carried out, including coronary angiography, disproved this suspicion.

As troponin was determined on the Abbott Architect i2000 immunoassay analyser, again with a sandwich technique using mouse antibodies, it could also have been a false positive result caused by heterophilic anti-mouse antibodies as in a similar case described in the literature (*28*).
